# Sex-based disparities in ascending aortic aneurysm surgery outcomes: a comprehensive analysis of 1148 consecutive patients with propensity-score matching

**DOI:** 10.1186/s13019-024-02646-6

**Published:** 2024-06-14

**Authors:** Mohammed Al-Tawil, Christine Friedrich, Alexandra Broll, Mohamed Salem, Jan Schoettler, Nora de Silva, Philipp Kolat, Felix Schoeneich, Assad Haneya

**Affiliations:** Department of Cardiovascular Surgery, University Hospital of Schleswig- Holstein, Campus Kiel, Kiel, Germany

**Keywords:** Ascending aortic aneurysm, Proximal aortic surgery, Aorta, Sex, Gender

## Abstract

**Background:**

Women undergoing cardiac surgery have been historically recognized to carry higher periprocedural mortality risk. We aimed to investigate the influence of sex on clinical presentation, perioperative, and long-term outcomes in patients who undergo surgery for ascending aortic aneurysm.

**Methods:**

We conducted a retrospective review of 1148 consecutive patients (380 [33.1%] female) who underwent thoracic aortic surgery under moderate hypothermic circulatory arrest for ascending aortic aneurysms between 2001 and 2021. Baseline and operative characteristics, in-hospital mortality, and survival were compared between male and female patients before and after propensity-score-matched (PSM) analysis.

**Results:**

Women were significantly older (median age: 69 [IQR: 63–75] vs. 67 [IQR: 58–73]; *P* < 0.001), while men had a higher prevalence of aortic valve stenosis, bicuspid valve and coronary artery disease at the time of surgery (*P* < 0.05). After PSM, EuroSCORE II (4.36 [2.68; 6.87] vs. 3.22 [1.85; 5.31]; *p* < 0.001), and indexed aortic diameter were significantly higher in female patients (2.94 [2.68; 3.30] vs. 2.58 [2.38; 2.81] cm/m2, *p* < 0.001). In the matched cohort, men were more likely to experience postoperative delirium (18.1% vs. 11.5%; *P* = 0.002), and postoperative neurological deficits (6.7% vs. 3.0%, *P* = 0.044),. Female patients were more likely to receive postoperative packed red blood cells (*p* = 0.036) and fresh frozen plasma (*p* = 0.049). In-hospital and 30-day mortality was similar between both groups. Long-term survival was comparable between both groups with 88% vs. 88% at 5 years, 76% vs. 71% at 10 years, and 59% vs. 47% at 15 years.

**Conclusion:**

Female patients required more transfusions, while males had a higher incidence of postoperative delirium and neurological deficits. Differences in preoperative age and timing of surgery between the sexes could be attributed to variations in comorbidity profiles and the greater prevalence of concomitant surgery indications in males.

**Supplementary Information:**

The online version contains supplementary material available at 10.1186/s13019-024-02646-6.

## Introduction

Women undergoing cardiac surgery have been historically underrepresented while being recognized to carry higher periprocedural mortality risk. The Society of Thoracic Surgeons (STS) and the EuroSCORE II risk prediction models weigh higher risk probabilities for female patients undergoing coronary bypass or valvular surgery [1–3]. Still, these scores did not establish the associated risk in women with ascending aortic aneurysms (AscAA).

It is established that the growth rate of thoracic aortic aneurysms is three-fold faster in women than in men, which has been theorized to be linked to hormonal changes in the menopausal period [4–7]. Such a faster growth rate is considered alarming, as it directly correlates with the risk for acute and catastrophic aortic events. Moreover, women with proximal aortic pathologies tend to have higher rates of life-threatening events and in-home mortality when compared to males [8]. The established threshold for surgical referral in patients with AscAA is a maximum aortic diameter > 55 mm [9, 10]. The current guidelines on diagnosis and management of aortic diseases do not define any sex-specific differences in AscAA, their progression rates, preoperative assessment, and long-term prognosis [9, 10].

Therefore, we aimed to investigate the influence of sex on clinical presentation, perioperative, and long-term outcomes in patients who undergo surgery for AscAA. While previous studies included a wider spectrum of patients, we tend to focus on presenting patients with AscAA rather than other ascending aortic diseases.

## Materials and methods

### Data acquisition and study design

We retrospectively reviewed patient data which was routinely collected in our institutional database for quality assurance. A total of 1148 consecutive patients who underwent aortic surgery for (AscAA) under moderate hypothermic circulatory arrest (MHCA) between 2001 and 2021 were identified. Patients who underwent surgery due to aortic dissection or proximal aortic calcifications were excluded.

We report and compare baseline patient characteristics and preoperative data between both sexes. The primary outcome was 30-day mortality. Secondary endpoints were length of intensive care unit (ICU) stay, length of hospital stay, postoperative stroke or delirium, postoperative drain output and all-cause survival.

### Surgical technique

The surgical technique used in our institution has been previously described [11, 12]. All surgeries were performed by senior faculty surgeons and experienced assistants. Aortic surgery was indicated at an ascending aortic diameter of more than 5.2 cm and over 4.5 cm in concomitant cardiac surgery in patients with no connective tissue disease. In patients with connective tissue disease, surgery was indicated at an ascending aortic diameter of 4.5-5.0 cm according to clinical evaluation.

All procedures were performed under general anaesthesia following median sternotomy, pericardiotomy, and standard cannulation for the instalment of cardiopulmonary bypass (CPB). In our institution, all distal aortic anastomoses were placed in MHCA at 24 °C. In complex cases with prolonged MHCA, we used selective antegrade cerebral perfusion.

Based on the coexisting valvular or coronary disease, further procedures including valve reconstruction/replacement and CABG were performed during rewarming from MHCA.

### Statistical analysis

We used descriptive statistics throughout the study to summarise baseline patient characteristics and procedural outcomes. Median and interquartile ranges (IQR) were used to describe normally and non-normally distributed continuous data. To test for group difference, we used Mann–Whitney’s U-test for non-normally distributed data and Student’s t-test for normally distributed data. Categorical variables are presented as frequency distributions (n) and simple percentages (%). Univariate comparison between the groups for categorical variables was made using the chi-square and Fisher’s exact tests.

Since preoperative findings and surgical techniques differed significantly between women and men, propensity score matching (PSM) based on the propensity score was conducted to analyze survival of female and male patients with homogeneous baseline characteristics. Propensity scores were calculated using multivariable logistic regression analysis with gender as dependent variable and age (year), body mass index (BMI), Ejection Fraction, aortic stenosis or regurge, bicuspid aortic valve, Marfan Syndrome, arterial hypertension, previous or current nicotine abuse, intubation at admission, coronary heart disease stage, chronic and decompensated renal insufficiency, pacemaker, previous PCI, previous CABG, supracoronary ascending aorta replacement, aortic root repair, additional Elephant-Trunk additional CABG, additional aortic or mitral valve replacement, and persisting foramen ovale closure as independent variables. We purposely did not include EuroSCORE into statistical matching since it contains gender itself as risk factor which would have biased matching. Matching was conducted pairwise with a maximum caliper width of 0.2 of the pooled standard deviation of the logit of the propensity score [13] Finally, 270 men and 270 women were matched and their main pre-, intra-, and postoperative findings are summarized in Tables (1, 2, 3, 4).

All tests were performed two-tailed and a *p*-value of 0.05 was considered statistically significant. Data were analyzed with IBM SPSS Statistics for Windows (Version 29.0).

## Results

### Preoperative data and baseline characteristics

Of the total cohort, 768 patients (66.9%) were men and 380 (33.1%) were women. Women were significantly older (median age: 69 [IQR: 63–75] vs. 67 [IQR: 58–73]; *P* < 0.001), had significantly larger aneurysm diameters (53 [IQR: 50–60] vs. 51 [IQR: 49–55] mm; *P* < 0.001), a higher aortic size index (2.99 [IQR: 2.72–3.33] vs. 2.54 [2.33–2.78]; *P* < 0.001), and a higher logistic EuroSCORE (23.3 [14.8;31.6] vs. 17.4 [10.3;26.4], *P* < 0.001) and EuroSCORE II (4.38 [2.82;6.70] vs. 3.57 [2.23;5.83], *P* < 0.001). Moreover, women were more likely to present with aortic regurgitation (49.0% vs. 38.1%; *P* < 0.001). Bicuspid aortic valve and aortic stenosis at the time of surgery were significantly higher in men (29,4% vs. 17.5%; *P* < 0.001; and 17.3% vs. 11.2%; *P* = 0.008, respectively). Further, men had more frequent coronary artery disease (42.6% vs. 28.9%; *P* < 0.001) and chronic renal insufficiency (10.8% vs. 5.8%; *P* = 0.006) and more frequent previously smokers (31.1% vs. 23.4%, *P* = 0.007).

After PSM, EuroSCORE II (4.36 [2.68; 6.87] vs. 3.22 [1.85; 5.31]; *p* < 0.001), and indexed aortic diameter were significantly higher in female patients (2.94 [2.68; 3.30] vs. 2.58 [2.38; 2.81] cm/m2, *p* < 0.001). Table 1 summarizes the baseline differences between both sexes prior to surgery. Table 2 illustrates preoperative differences in laboratory data.

### Intraoperative details

In terms of intraoperative data, men were more likely to undergo concomitant CABG (29.8% vs. 16.7%; *P* < 0.001) with more distal anastomoses (2 [1;3] vs. 1 [1;2], *P* = 0.005), aortic root repair (22.9% vs. 15.6%; *P* < 0.004), and aortic valve replacement (57.5% vs. 45.8%; *P* < 0.001).

After PSM, women in our cohort had significantly higher rates of intraoperative packed red blood cell transfusion (pRBCs) (2 [IQR:1–14] vs. 1 [IQR: 0–18] units; *P* < 0.001) and larger valve prosthesis size (27 [25;27] vs. 23 [23;25], *P* < 0.001). Operative time, bypass time, and aortic cross-clamp time were significantly longer in men when compared to women. However, the durations were comparable after PSM. See Table 3 which outlines the intraoperative details of patients in our cohort.

### Postoperative outcomes

In our PSM cohort, there was no difference between both genders in terms of in-hospital mortality (3.3% vs. 3.7%; *P* = 0.80), or 30-day mortality (3.4% vs. 3.8%; *P* = 0.78) in the male and female group. No major difference was observed in terms of reintervention, or ICU length of stay between the two groups. However, men were more likely to experience postoperative delirium (18.1% vs. 11.5%; *P* = 0.002), and postoperative neurological deficits (6.7% vs. 3.0%, *P* = 0.044). Female patients were more likely to receive postoperative pRBCs (*p* = 0.036) and fresh frozen plasma (*p* = 0.049). Table 4 summarizes postoperative data for the entire cohort. Table 5 illustrates cause-specific in-hospital and 30-day mortality.

### Follow-up

**Fig. 1:**
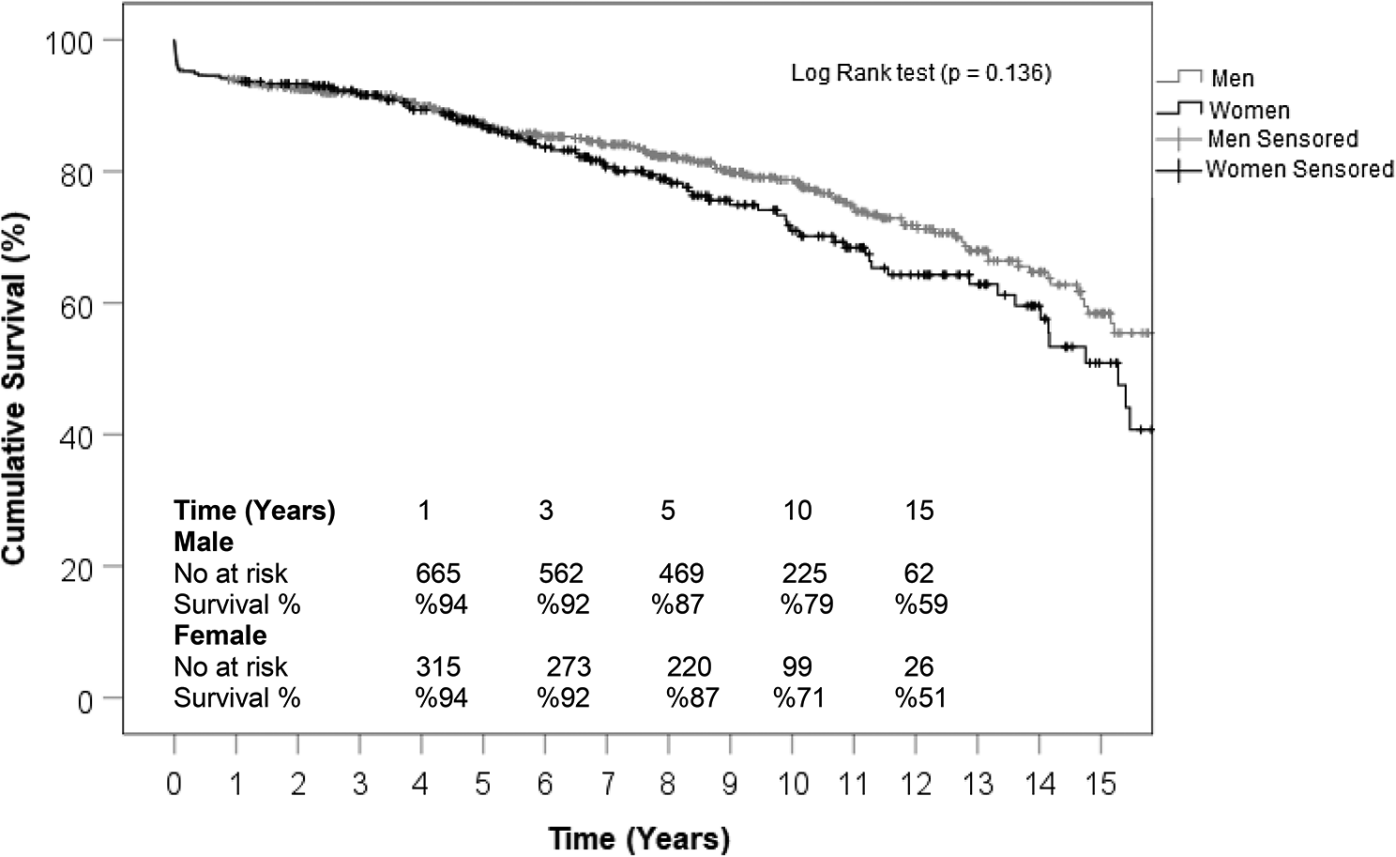
Kaplan-Meier Curve illustrating long-term survival throughout follow-up for 85.5% of patients

**Figure 2:**
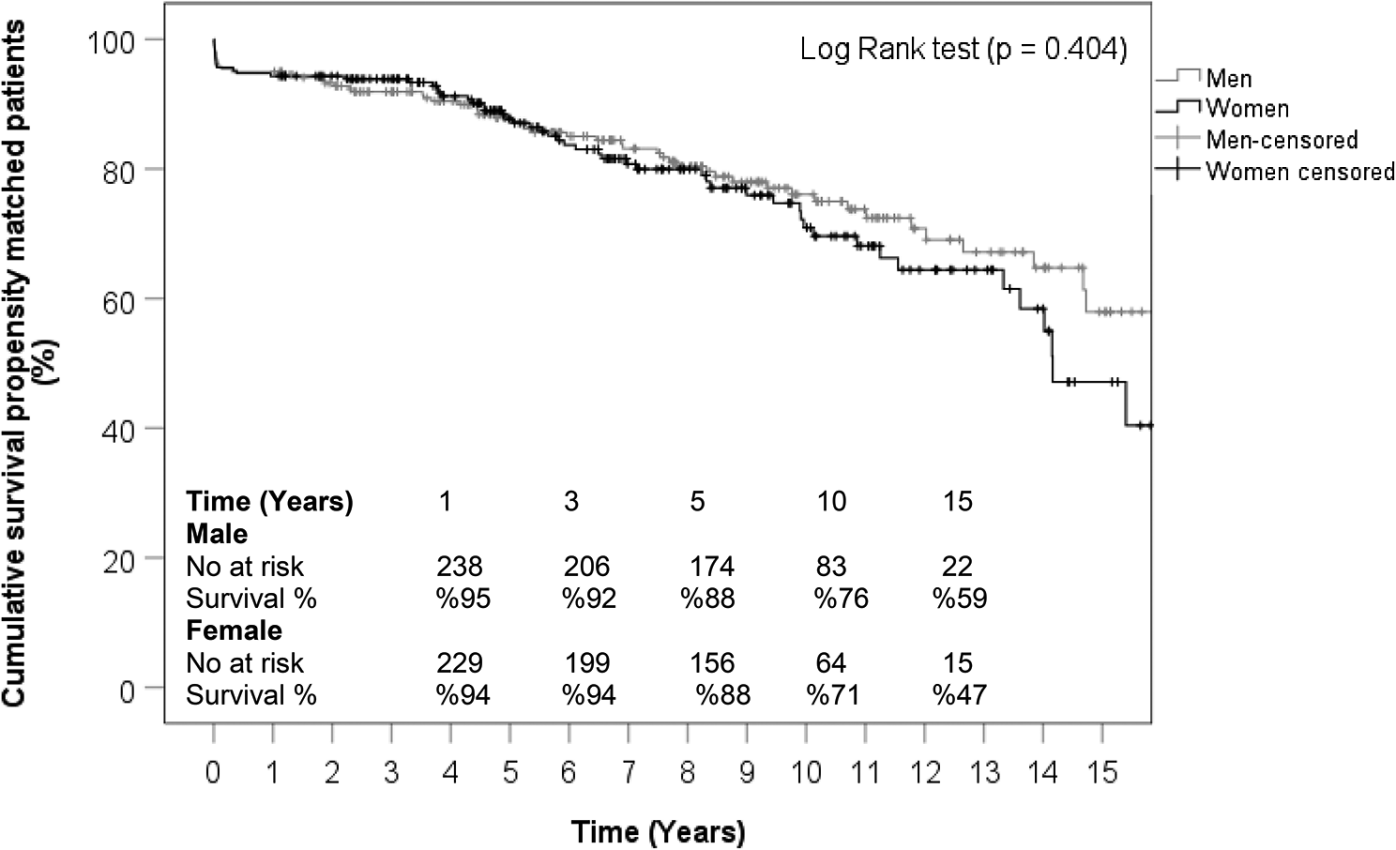
Kaplan-Meier Curve illustrating long-term survival throughout follow-up for propensity matched patients

**Figure 3:**
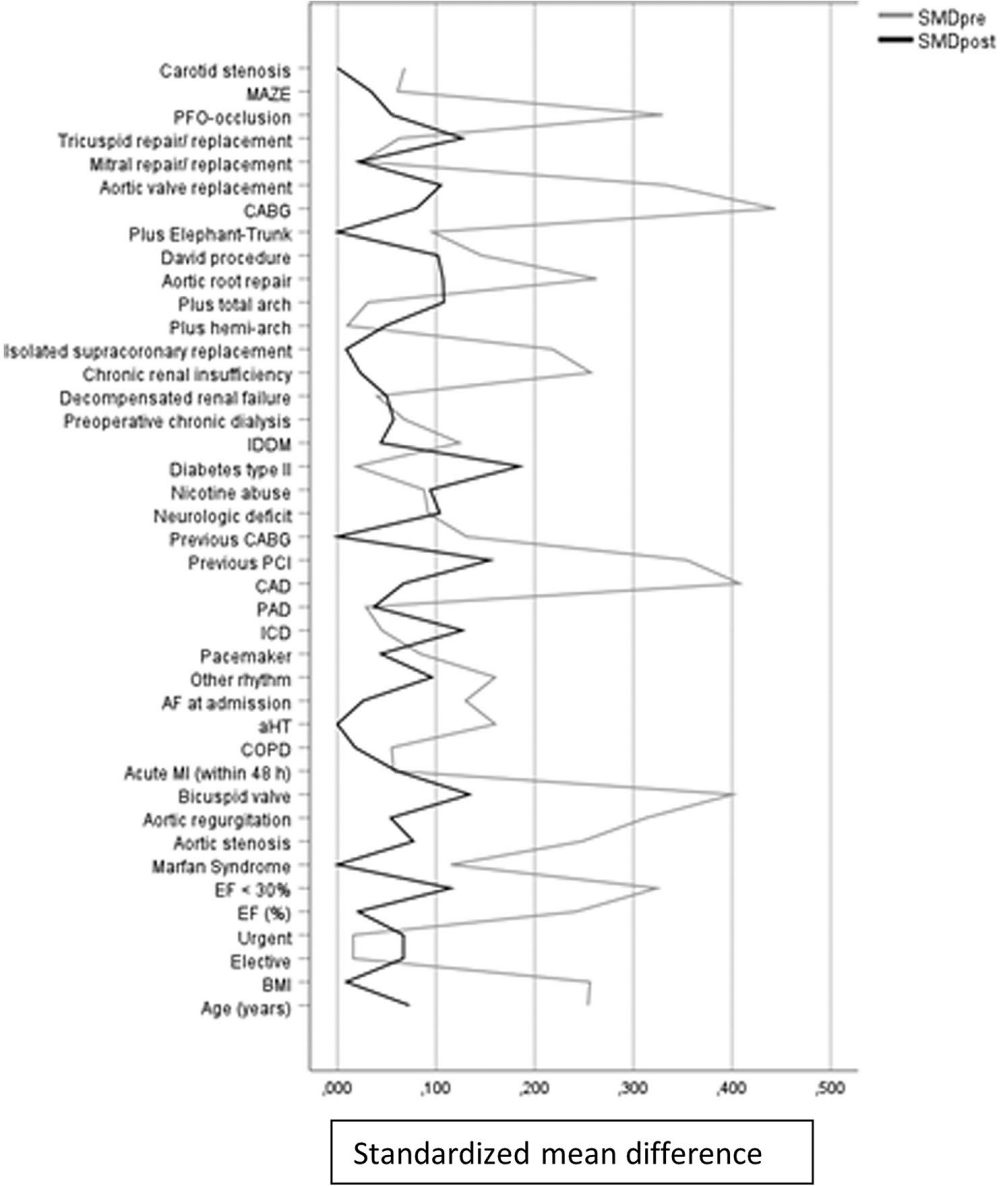
Love plot displaying the standardized mean difference between the male and female groups pre- and post- matching

Long-term follow-up was available for 85.5% of the entire cohort with a median of 7.1 years [IQR: 3.9–10.8]. There was no significant difference between both sexes in median follow-up time (Men: 7.3 [IQR: 4.0-10.8] years vs. Women 6.7 [IQR: 3.8–10.8]; *P* = 0.6). Actuarial survival was similar between both groups with 88% vs. 88% at 5 years, but numerically higher in men with 76% vs. 71% at 10 years, and 59% vs. 47% at 15 years. However, the results did not reach statistical significance. Kaplan-Meier curve illustrates survival probabilities for both sexes before and after PSM are illustrated in Fig. 1 and Fig. 2, respectively. Figure 3 displays a love plot which shows the standardized mean difference before and after PSM.

## Discussion

Our analysis represents a large, single-center series of patients who underwent ascending aortic surgery for AscAA between 2001 and 2021. At the time of surgery, women were older, had significantly larger aneurysmal diameters, higher EuroSCOREs, had more frequent aortic regurgitation, were more often intubated prior to surgery and had higher left ventricular ejection fraction. On the other hand, men were younger and had more frequent bicuspid aortic valves, aortic stenosis, coronary artery disease, and chronic renal insufficiency.

After PSM, only the EuroSCORE, and the indexed aortic diameter were significantly higher in female patients.

Similar to recently published data [8, 14–17], women are more likely to present later in life, with a significantly larger indexed aortic diameter or absolute aneurysm diameter. This is crucial in highlighting the seriousness of aneurysmal progression in women and their underestimated risk in the current practice. The observed differences in indexed aortic diameter in our cohort support the notion that while females have smaller aortas, the onset of the disease maybe later in life after the menopause, and the nature of the progression maybe more incipient when compared to their male counterparts. This can be further supported by the fact the male patients have more cardiovascular comorbidities and therefore, they might have undergone more regular medical evaluation. These findings emphasize the need for increased awareness and tailored management strategies for women with AscAA.

Historically, female patients had higher cardiovascular risk and later presentation with more advanced disease [18–20]. It is unclear, however, whether female patients have comparable outcome profiles to their male counterparts after AscAA repair.

For patients with no elastopathies, the AscAA threshold for surgery has been established at a diameter of 55 mm [9, 10]. In light of recently published data and risk stratification studies, there was an evident trend toward lowering the threshold for AscAA surgery to 50 mm [10]. Nonetheless, few reports advocate for indexing aortic diameter to body surface area or patient’s height, which has been recommended only for patients who are significantly taller or shorter than average [10, 21]. Such differences are not accounted for in current practice and cannot be explored by analysis of patients who actually undergo the surgery.

As their surgery is indicated later in the disease course, published results [14, 15] demonstrated a higher tendency for arch involvement in women. In contrast, we did not observe any difference between both sexes in terms of indicated extended arch replacement. In line with Beller’s and Voigt’s results [14, 15], an isolated supra-coronary aortic replacement was more frequent in women in our cohort, while men tended to undergo more frequent aortic root replacement.

Similar to intraoperative data of other centres [8, 15, 17], men in our cohort had consistently longer operative, cardiopulmonary bypass, and/or aortic cross-clamp times. This difference disappeared after controlling for concomitant procedures, which are indicated more frequently in men across studies such as concomitant CABG as in our male subgroup due to a greater incidence of coronary artery disease or valve replacement [8, 15, 17]. Despite their comparable operation times and less complex surgery, women received more intraoperative blood transfusions with significant difference after PSM. Several studies reported higher transfusion rates, lower preoperative haemoglobin or haematocrit levels prior to cardiovascular surgery and a higher degree of haemodilution on cardiopulmonary bypass in women compared to men [22–25], which may explain this intraoperative discrepancy in our study. It`s noteworthy that Mehta et al. [24] in a large analysis on 13,739 patients undergoing cardiac surgery, suggest that women have a better tolerance to haemodilution and that specific thresholds for blood transfusions in women may reduce its harmful effects.

Men developed a postoperative delirium and more neurological deficits. This is in line with the findings of Wang et al. [26] showing that male gender is an important predictor for postoperative delirium following cardiac surgery.

In our large cohort, we did not observe differences in mortality rates following surgery or in long-term survival between both sexes. Still, Kampen and colleagues [8] highlighted a significant four-fold higher in-house mortality in women. Such a finding emphasizes the consequence of the delayed indication for surgery in the women subpopulation.

Parallel to our results, in-hospital and short-term mortality following proximal aortic surgery was similar between both sexes in most of the published results [14–16, 22]. However, long-term survival was inconsistent in the literature, with some studies showing significantly lower survival in women [8, 14, 15], while others reporting no significant difference between both sexes [16, 22, 27]. Patients in our series had similar survival probability throughout and at 15 years of follow-up. In studies that included only patients with AscAA [8, 14, 15], Kaplan Meier analysis showed significantly lower long-term survival in women when compared to men. In our analysis, women tended to have lower long-term survival, Fig. 1, however, the results did not reach statistical significance.

The previously mentioned data regarding the relationship between sex and survival after proximal aortic surgery point out the higher risk in women, following the same pattern in other cardiovascular surgeries [18–20]. Nonetheless, it also encourages a deeper investigation into the specific preoperative characteristics of women, that could impact their preoperative screening, assessment, and surgery planning.

## **Limitations**

Our study has several limitations, one is the observational and retrospective nature of data retrieved from our institution’s registry, thus, allowing for bias from unmeasured confounders. We only analyzed patients who underwent the surgery, patients who offered the surgery and refused were not incorporated in the pre-operative data. Further, we could not perform a retrograde analysis to detect pre-hospital events. The limited spectrum of data available solely from patients during their in-hospital stay poses a limitation in providing a comprehensive overview of their overall status and the course of the disease. Our study primarily focused on examining the intra and postoperative courses of disease between the two sexes, which provides valuable insights. However, the lack of preoperative extensive follow-up data restricts our ability to fully capture the pre-hospital outcomes, including non-operated patients or those who experienced death prior to hospital admission. Another important limitation is the long duration of our study, which lead varying definitions/ cutoffs used to diagnose comorbidities and postoperative complications. We acknowledge the importance of further research to explore disease profiles in both sexes.

## Conclusions

In our large analysis, we could demonstrate that women are older and have more advanced disease when indicated for surgery. We found that these differences could be largely attributed to variations in the distribution of comorbidities between the two groups that necessitate concomitant interventions. After PSM,emale patients required more transfusions andmale patients exhibited a higher incidence of delirium and postoperative neurological deficits following surgery. There were no significant differences in short-term or long-term survival between men and women. The observed disparities in age and timing of surgery between the sexes in the overall cohort can be largely attributed to differences in the morbidity profile and the need for concomitant procedures which are more frequently observed in males.

**Table 1:** Baseline Characteristics and Preoperative data

Parameter	Overall Cohort			After Propensity-Score Matching		
	Male *n* = 768; 66.9%	Female *n* = 380; 33,1%	*P* value	Male *n* = 270; 50%	Female *n* = 270; 50%	*P* value
Age	67 (58; 73)	69 (63; 75)	**< 0.001**	69 (61; 74)	69 (62; 74)	0.554
Weight (kg)	85 (78; 95)	70 (62; 80)	**< 0.001**	84 (75; 94)	72 (63; 81)	**< 0.001**
Height (cm)	178 (174; 183)	166 (161; 170)	**< 0.001**	178 (173; 183)	166 (160; 170)	**< 0.001**
BSA (m^2^)	2.04 (1.94; 2.16)	1.79 (1.67; 1.89)	**< 0.001**	2.02 (1.93; 2.14)	1.81 (1.68; 1.91)	**< 0.001**
BMI	26.8 (24.6; 29.6)	25.3 (23.0; 28.9)	**< 0.001**	26.5 (24.3;29.1)	25.8 (23.4; 29.7)	0.431
BMI > 30	172 (22.4%)	73 (19.3%)	0.227	53 (19.6%)	64 (23.7%)	0.251
Aneurysm diameter (mm)	51 (49; 55)	53 (50; 60)	**< 0.001**	52 (50; 55)	52 (50; 59)	0.190
Aortic size Index (cm/ m^2^)	2.54 (2.33; 2.78)	2.99 (2.72; 3.33)	**< 0.001**	2.58 (2.38; 2.81)	2.94 (2.68; 3.30)	**< 0.001**
Logistic EuroScore	17.36 (10.29; 26.44)	23.27 (14.78;31.56)	**< 0.001**	15.90 (10.21; 25.35)	21.48 (12.89;31.67)	**< 0.001**
EuroScore II	3.57 (2.23; 5.83)	4.38 (2.82; 6.70)	**< 0.001**	3.22 (1.85; 5.31)	4.36 (2.68; 6.87)	**< 0.001**
Elective	745 (97.0%)	368 (96.8%)	0.880	263 (97.4%)	265 (98.1%)	0.559
Urgent	23 (3.0%)	12 (3.2%)	0.880	7 (2.6%)	5 (1.9%)	0.559
EF %,	60 (50; 70)	65 (55; 70)	**< 0.001**	65 (55; 70)	64 (55; 70)	0.837
EF < 30	28 (3.8%)	2 (0.5%)	**0.002**	3 (1.1%)	1 (0.4%)	0.624
Marfan Syndrome	13 (1.7%)	3 (0.8%)	0.221	3 (1.1%)	3 (1.1%)	1.000
Aortic stenosis	126 (17.3%)	41 (11.2%)	**0.008**	41 (15.2%)	36 (13.3%)	0.538
Aortic regurgitation	278 (38.1%)	180 (49.0%)	**< 0.001**	129 (47.8%)	124 (45.9%)	0.666
Bicuspid valve	136 (29,4%)	38 (17.5%)	**< 0.001**	119 (77.8%)	124 (81.6%)	0.409
Acute MI (within 48 h)	9 (1.2%)	3 (0.8%)	0.760	2 (0.7%)	3 (1.1%)	1.000
Intubated at admission	1 (0.1%)	4 (1.1%)	**0.044**	0 (0.0%)	0 (0.0%)	----
COPD	76 (9.9%)	42 (11.1%)	0.548	29 (10.7%)	30 (11.1%)	0.890
aHT	569 (74.1%)	299 (78.9%)	0.075	212 (78.5%)	212 (78.5%)	1.000
AF on admission	148 (19.3%)	60 (15.8%)	0.150	47 (17.4%)	45 (16.7%)	0.819
Pacemaker	26 (3.4%)	9 (2.4%)	0.349	4 (1.5%)	5 (1.9%)	1.000
ICD	4 (0.5%)	1 (0.3%)	1.000	0 (0.0%)	1 (0.4%)	1.000
Other rhythm	58 (7.6%)	18 (4.7%)	0.072	12 (4.4%)	16 (5.9%)	0.438
PAD	29 (3.8%)	16 (4.2%)	0.724	6 (2.2%)	7 (2.6%)	0.779
CAD	326 (42.6%)	110 (28.9%)	**< 0.001**	84 (31.1%)	90 (33.3%)	0.581
1-vessel disease	142 (18.6%)	67 (17.6%)	**0.701**	54 (20.0%)	54 (20.0%)	1.000
2-vessel disease	71 (9.3%)	25 (6.6%)	**0.120**	22 (8.1%)	20 (7.4%)	0.748
3-vessel disease	113 (14.8%)	18 (4.7%)	**< 0.001**	8 (3.0%)	16 (5.9%)	0.095
Previous PCI	73 (9.5%)	13 (3.4%)	**< 0.001**	5 (1.9%)	10 (3.7%)	0.190
Previous CABG	18 (2.3%)	4 (1.1%)	0.133	2 (0.7%)	2 (0.7%)	1.000
Cardiac catheterization	731 (95.4%)	350 (92.3%)	**0.033**	262 (97.0%)	252 (93.7%)	0.064
PreOP neurologic deficits	83 (10.8%)	49 (12.9%)	0.300	24 (8.9%)	30 (11.1%)	0.397
Nicotine abuse	143 (18.8%)	78 (21.3%)	0.339	48 (17.8%)	55 (20.4%)	0.443
Previous nicotine abuse	236 (31.1%)	86 (23.4%)	0.007	73 (27.0%)	71 (26.3%)	0.846
Diabetes Type II	74 (9.6%)	35 (9.2%)	0.817	19 (7.0%)	29 (10.7%)	0.130
IDDM	19 (2.5%)	5 (1.3%)	0.197	5 (1.9%)	4 (1.5%)	1.000
Preop chronic dialysis	7 (0.9%)	2 (0.5%)	0.726	2 (0.7%)	4 (0.4%)	1.000
Decompensated renal failure	8 (1.0%)	5 (1.3%)	0.768	3 (1.1%)	4 (1.5%)	1.000
Chronic renal insufficency	83 (10.8%)	22 (5.8%)	**0.006**	18 (6.7%)	17 (6.3%)	0.861

**Table 2:** Preoperative laboratory parameters

Parameter	Overall Cohort			After Propensity-Score Matching		
	Male *n* = 768; 66.9%	Female *n* = 380; 33.1%	*P* value	Male *n* = 270; 50%	Female *n* = 270; 50%	*P* value
Hematocrit	42 (39;44)	39 (36;41)	**< 0.001**	42 (39;44)	39 (36;41)	**< 0.001**
Hb (g/dl)	14.4 (13.4;15.2)	13.1 (12.1;13.9)	**< 0.001**	14.3 (13.5;15.0)	13.1 (12.1;14.0)	**< 0.001**
Lactate (mmol/l)	1.6 (1.1;2.2)	1.9 (1.2;2.7)	**0.002**	1.5 (1.0;2.2)	1.8 (1.1;2.7)	**0.003**
Sodium (mmol/l)	137 (135;139)	137 (135;139)	0.919	137 (135;139)	137 (135;139)	0.715
Potassium (mmol/l)	3.8 (3.6;4.1)	3.7 (3.5;4.0)	**< 0.001**	3.8 (3.6;4.0)	3.7 (3.5;4.0)	**0.019**
Creatine kinase (IU)	93 (65;141)	77 (54;161)	**< 0.001**	95 (68;150)	76 (53;109)	**< 0.001**
Creatinine (mg/dl)	1.00 (0.87;1.17)	0.82 (0.72;0.94)	**< 0.001**	85 (74; 98)	72 (64;83)	**< 0.001**
Urea (mmol/l)	5.85 (4.80;7.20)	5.60 (4.33;6.90)	**0.004**	5.83 (4.70;7.31)	5.51 (4.33;6.70)	**0.025**
GFR (ml/min)	61 (60;75)	61 (60;70)	**0.049**	61 (60;75)	61 (60;71)	0.087
GPT (IU)	24 (17;33)	18 (14;24)	**< 0.001**	23 (17;33)	19 (14;24)	**< 0.001**
INR	1.03 (0.98;1.10)	1.01 (0.96;1.08)	**< 0.001**	1.03 (0.97;1.09)	1.01 (0.96;1.07)	**0.010**
CRP (mg/l)	2.2 (0.9;5.4)	2.9 (1.2;6.7)	**0.007**	2.3 (0.9;5.3)	2.9 (1.2;6.5)	0.080
Leukocytes (109/L)	6.91 (5.76;8.35)	6.89 (5.76;8.22)	0.697	6.75 (5.56;8.08)	6.87 (5.76;8.24)	0.440
Platelets (109/L)	211 (178;250)	236 (194;282)	**< 0.001**	203 (178;237)	237 (195;278)	**< 0.001**

**Table 3:** Intraoperative details

Parameter	Overall Cohort			After Propensity-Score Matching		
	Male *n* = 768; 66.9%	Female *n* = 380; 33.1%	*p* value	Male *n* = 270; 50%	Female *n* = 270; 50%	*p* value
Surgery duration (min)	265 (215; 323)	231 (196; 288)	**< 0.001**	244 (197;305)	234 (200; 290)	0.546
CPB (min)	157.5 (122; 195)	132 (107;181)	**< 0.001**	141 (112; 186)	134 (108;180)	0.214
Aortic cross clamp (min)	106 (76.5; 138)	86 (58; 116)	**< 0.001**	93 (62; 133)	90 (60; 118)	0.207
Circulatory arrest (min)	14 (12;17)	15 (13; 19)	0.069	14 (12;17)	15 (13; 19)	**0.019**
RBC intraop (unit)	1 (0;2)	2 (1;4)	**< 0.001**	1 (0–18)	2 (0–14)	**< 0.001**
FFP intraop (unit)	0 (0;0)	0 (0;1)	0.285	0 (0–14)	0 (0–16)	0.533
Platelets intraop (unit)	1 (0; 2)	1 (0;1)	0.365	1 (0–5)	1 (0–8)	0.938
Isolated supracoronary replacement	505 (65.8%)	277 (72.9%)	**0.015**	190 (70.4%)	191 (70.7%)	0.925
Plus Hemi-arch	198 (25.8%)	99 (26.1%)	0.921	70 (25.9%)	66 (24.4%)	0.692
Plus total arch	25 (3.3%)	14 (3.7%)	0.700	13 (4.8%)	9 (3.3%)	0.389
Aortic root repair	176 (22.9%)	59 (15.6%)	**0.004**	57 (21.1%)	49 (18.1%)	0.386
David procedure	60 (7.8%)	41 (10.8%)	0.091	31 (11.5%)	25 (9.3%)	0.397
Plus Elephant-Trunk	8 (1.0%)	7 (1.8%)	0.276	5 (1.9%)	5 (1.9%)	1.000
CABG	229 (29.8%)	63 (16.7%)	**< 0.001**	48 (17.8%)	54 (20.0%)	0.509
Number of distal anstomoses	2 (1;3)	1 (1;2)	**0.005**	1 (1;2)	1 (1;2)	0.883
Aortic valve replacement	441 (57.5%)	174 (45.8%)	**< 0.001**	142 (52.6%)	132 (48.9%)	0.389
Size of Aortic valve prosthesis (mm)	27 (25;27)	23 (23;25)	**< 0.001**	27 (25;27)	23 (23;25)	**< 0.001**
Mitral repair/replacement	22 (2.9%)	12 (3.2%)	0.777	12 (4.4%)	11 (4.1%)	0.831
Tricuspid repair/replacement	1 (0.1%)	1 (0.3%)	0.553	0 (0.0%)	1 (0.4%)	1.000
PFO-occlusion	57 (7.4%)	9 (2.4%)	**< 0.001**	10 (3.7%)	8 (3.0%)	0.632
Maze	24 (3.1%)	9 (2.4%)	0.464	8 (3.0%)	7 (2.6%)	0.782
Carotid stenosis	7 (0.9%)	3 (0.5%)	0.726	2 (0.7%)	2 (0.7%)	1.000

**Table 4:** Postoperative data

Parameter	Overall Cohort			After Propensity-Score Matching		
	Male *n* = 768; 66.9%	Female *n* = 380; 33.1%	*P* value	Male *n* = 270; 50%	Female *n* = 270; 50%	*P* value
Acute kidney injury	50 (6.5%)	16 (4.2%)	0.115	12 (4.4%)	13 (4.8%)	0.838
Post OP dialysis/ hemofiltration	39 (5.1%)	15 (4.0%)	0.390	12 (4.5%)	11 (4.1%)	0.824
Re-intubation	69 (9.0%)	21 (5.5%)	0.247	25 (9.3%)	19 (7.0%)	0.345
Tracheotomy	54 (7.1%)	16 (4.2%)	0.061	16 (5.9%)	8 (3.0%)	0.099
ICU re-admission	43 (5.6%)	19 (5.0%)	0.673	15 (5.6%)	17 (6.3%)	0.715
Delirium	128 (16.7%)	38 (10.0%)	**0.002**	49 (18.1%)	31 (11.5%)	**0.029**
New neurologic deficits cCT confirmed	48 (6.3%)	14 (3.7%)	0.070	18 (6.7%)	8 (3.0%)	**0.044**
PostOP CPR	18 (2.3%)	11 (2.9%)	0.703	4 (1.5%)	8 (3.0%)	0.243
AF on discharge	108 (14.2%)	47 (12.5%)	0.415	38 (14.2%)	37 (13.8%)	0.887
Post Op pacemaker	54 (7%)	15 (4%)	**0.039**	15 (5.6%)	11 (4.1%)	0.427
Myocardial Infarction PostOP	3 (0.4%)	2 (0.5%)	0.668	1 (0.4%)	2 (0.7%)	1.000
Pneumonia	65 (8.5%)	19 (5.0%)	**0.034**	16 (5.9%)	16 (5.9%)	1.000
Sepsis	24 (3.1%)	7 (1.8%)	0.206	11 (4.1%)	5 (1.9%)	0.128
Chest drain output 48 h (ml)	600 (400; 1000)	550 (350; 925)	**0.004**	600 (400; 900)	550 (350; 980)	0.255
Pericardial tamponade	31 (4.2%)	8 (2.2%)	0.214	12 (4.5%)	9 (3.4%)	0.515
Rethoracotomy	57 (7.4%)	27 (7.1%)	0.846	20 (7.4%)	21 (7.8%)	0.871
Reintervention TEVAR	1 (0.1%)	2 (0.5%)	0.256	1 (0.4%)	1 (0.4%)	1.000
Wound healing deficits/VAC Revision	11 (1.4%)	3 (0.8%)	0.409	4 (1.5%)	3 (1.1%)	1.000
RBC 24 h postop	0 (0;2)	0 (0;2)	0.199	0 (0–11)	0 (0–14)	**0.036**
FFPs 24 h postop	0 (0;2)	0 (0;3)	0.565	0 (0–15)	0 (0–23)	**0.049**
Platelets 24 h postop	0 (0;0)	0 (0;0)	0.696	0 (0–5)	0 (0–7)	0.676
Ventilation (h)	16 (11; 26)	17 (12;26)	0.108	16 (10; 24)	17 (12;28)	0.071
ICU stay (d)	2 (1; 4)	2 (1;4)	0.580	2 (1; 4)	2 (1;4)	0.203
PostOP Days	9 (8; 13)	9 (7;13)	0.873	9 (7; 13)	9 (7;13)	0.929
Surgery till death (d)	11 (5;19)	8 (3;18)	0.558	14 (6;22)	8 (4;13)	0.417

**Table 5:** Mortality and follow-up data

Parameter	Overall Cohort			After Propensity-Score Matching		
	Male *n* = 768; 66.9%	Female *n* = 380; 33.1%	*P* value	Male *n* = 270; 50%	Female *n* = 270; 50%	*P* value
In-hospital mortality	26 (3,4%)	14 (3,7%)	0.795	9 (3.3%)	10 (3.7%)	0.809
Cardiac	10 (38,5%)	8 (57,1%)	0.499	5 (55.6%)	5 (50.0%)	0.800
Cerebral	4 (14,5%)	0 (0,0%)	0.499	1 (11.1%)	0 (0.0%)	0.800
Sepsis	3 (11,5%)	1 (7,1%)	0.499	1 (11,1%)	1 (10.0%)	0.800
MOF	9 (34,6%)	5 (35,7%)	0.499	2 (22.2%)	4 (40.0%)	0.800
7-day Mortality	12 (1.6%)	8 (2.1%)	0.508	4 (1.5%)	5 (1.9%)	0.752
30-day Mortality	28 (3.7%)	15 (4%)	0.787	9 (3.4%)	10 (3.8%)	0.802
Cardiac	9 (32,1%)	8 (53,3%)	0.425	4 (44.4%)	5 (50.0%)	1.000
Cerebral	4 (14,3%)	0 (0,0%)	0.425	1 (11.1%)	0 (0.0%)	1.000
Sepsis	2 (7,1%)	1 (6,7%)	0.425	1 (11.1%)	1 (10.0%)	1.000
MOF	11 (39,3%)	4 (26,7%)	0.425	3 (33.3%)	3 (30.0%)	1.000
Unknown	2 (7,1%)	2 (13,3%)	0.425	0 (0.0%)	1 (10.0%)	1.000
Follow-up						
Follow-up completeness	667 (86.8%)	315 (82.9%)	0.073	238 (88.1%)	229 (84.8%)	0.254
Follow-up in years	7.3 (4.0;10.8)	6.7 (3.8; 10.8)	0.623	7.7 (4.2;10.8)	6.5 (3.8; 9.9)	0.110

### Electronic supplementary material

Below is the link to the electronic supplementary material.

Supplementary Material 1

## Data Availability

The data analyzed in this study is subject to the following licenses/restrictions: Institutional dataset. Reasonable requests to access these datasets should be directed to the corresponding author.

## Abbreviations

STS: Society of Thoracic Surgeons
AscAA: ascending aortic aneurysms
MHCA: moderate hypothermic circulatory arrest
ICU: intensive care unit
CPB: Cardiopulmonary bypass
CABG: Coronary Artery Bypass Grafting
IQR: Interquartile Range
PSM: Propensity Score Matching
BMI: Body Mass Index
PCI: Percutaneous Coronary Intervention
