# Supplementary material for: Sex-based disparities in ascending aortic aneurysm surgery outcomes: a comprehensive analysis of 1148 consecutive patients with propensity-score matching
Source: J Cardiothorac Surg. 2024 Jun 14;19:331. doi: 10.1186/s13019-024-02646-6 (PMC11177366; doi:10.1186/s13019-024-02646-6)
Supplement: Supplementary file 1 — Supplementary Material 1 [file 13019_2024_2646_MOESM1_ESM.docx]

**Supplementary Material File**

**
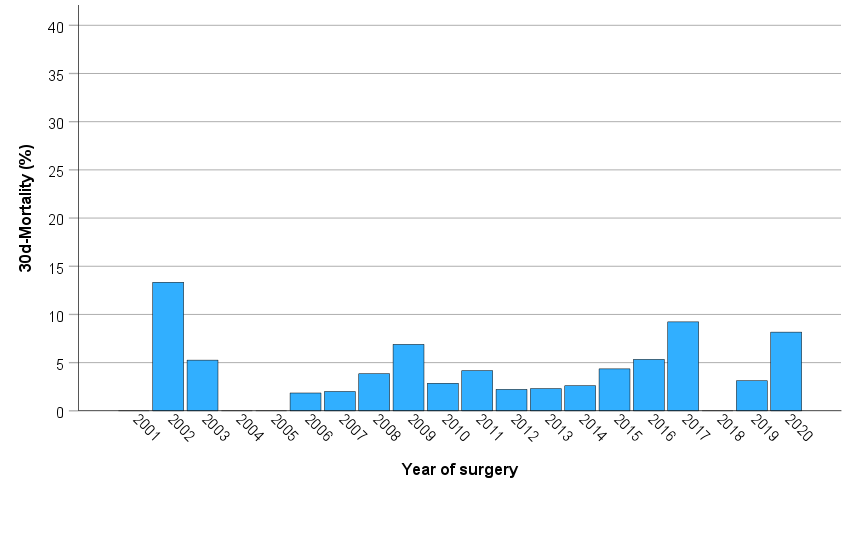
**

**Figure (S1): Chronological overview of 30-days mortality rates over the last 19 years of surgery at our institution.**

**___________________________________________________________________**

**Table (S2): Standardized mean difference (SMD) pre- and post-matching:**

| **Parameter** | SMD pre-matching | SMD post-matching |
| --- | --- | --- |
| Age (years) | 0.254 | 0.073 |
| Weight (kg) | 1.084 | 0.845 |
| Height (cm) | 1.876 | 1.817 |
| BSA (m2) | 1.536 | 1.284 |
| BMI | 0.256 | 0.009 |
| BMI >30 | 0.108 | 0.141 |
| Aneurysm diameter (mm) | 0.303 | 0.216 |
| Aortic size Index (cm/ m^2^) | 0.950 | 0.807 |
| Logistic EuroScore | 0.335 | 0.341 |
| EuroScore II | 0.165 | 0.231 |
| Elective | 0.016 | 0.067 |
| Urgent | 0.016 | 0.067 |
| EF % | 0.242 | 0.021 |
| EF<30 | 0.324 | 0.115 |
| Marfan Syndrome | 0.115 | 0.000 |
| Aortic stenosis | 0.248 | 0.077 |
| Aortic regurgitation | 0.313 | 0.054 |
| Bicuspid valve | 0.401 | 0.134 |
| Acute MI (within 48h) | 0.057 | 0.060 |
| COPD | 0.055 | 0.018 |
| aHT | 0.160 | 0.000 |
| AF at admission | 0.130 | 0.026 |
| Other rhythm | 0.160 | 0.096 |
| Pacemaker | 0.084 | 0.044 |
| ICD | 0.045 | 0.127 |
| PAD | 0.029 | 0.037 |
| CAD | 0.408 | 0.067 |
| 1-vessel disease | 0.037 | 0.000 |
| 2-vessel disease | 0.141 | 0.037 |
| 3-vessel disease | 0.489 | 0.199 |
| Previous PCI | 0.354 | 0.155 |
| Previous CABG | 0.131 | 0.000 |
| Cardiac catheterization | 0.183 | 0.222 |
| Preoperative neurologic deficits | 0.092 | 0.104 |
| Nicotine abuse | 0.088 | 0.094 |
| Previous nicotine abuse | 0.245 | 0.022 |
| Diabetes Type II | 0.019 | 0.185 |
| IDDM | 0.124 | 0.044 |
| Preop chron dialysis | 0.068 | 0.057 |
| Decompensated renal failure | 0.040 | 0.050 |
| Chron renal insufficiency | 0.257 | 0.023 |
| Surgery duration (min) | 0.310 | 0.046 |
| CPB (min) | 0.274 | 0.090 |
| Aortic cross clamp (min) | 0.326 | 0.111 |
| Circulatory arrest (min) | 0.050 | 0.006 |
| RBC (unit) | 0.357 | 0.368 |
| FFP (unit) | 0.021 | 0.004 |
| Platelets (unit) | 0.043 | 0.021 |
| Isolated supracoronary replacement | 0.218 | 0.009 |
| Plus hemi-arch | 0.010 | 0.049 |
| Plus total arch | 0.031 | 0.108 |
| Aortic root repair | 0.263 | 0.107 |
| David procedure | 0.146 | 0.102 |
| Plus Elephant-Trunk | 0.096 | 0.000 |
| CABG | 0.444 | 0.080 |
| Number of distal anstomosis | 0.407 | 0.028 |
| Aortic valve replacement | 0.333 | 0.105 |
| Size of aortic valve prosthesis (mm) | 1.267 | 1.265 |
| Mitral repair/replacement | 0.025 | 0.021 |
| Tricuspid repair/replacement | 0.063 | 0.127 |
| PFO-occlusion | 0.330 | 0.055 |
| Maze | 0.061 | 0.034 |
| Carotid stenosis | 0.068 | 0.000 |
